# Effect of planecta and ROSE™ on the frequency characteristics of blood pressure-transducer kits

**DOI:** 10.1007/s10877-014-9650-y

**Published:** 2014-12-17

**Authors:** Shigeki Fujiwara, Yoshifumi Kawakubo, Satoshi Mori, Keiichi Tachihara, Izumi Toyoguchi, Takeshi Yokoyama

**Affiliations:** 1 Department of Dental Anesthesiology, Faculty of Dental Science, Kyushu University, 3-1-1 Maidashi, Higashi-ku, Fukuoka, 812-8582 Japan; 2 Argon Medical Devices Japan, Davinchi-Ningyocho 6F, 2-13-9 Nihonbashi-ningyochou, Chuo-ku, Tokyo, 103-0013 Japan

**Keywords:** Frequency characteristics, Planecta, ROSE, Blood pressure-transducer kits

## Abstract

Pressure-transducer kits have frequency characteristics such as natural frequency and damping coefficient, which affect the monitoring accuracy. The aim of the present study was to investigate the effect of planecta ports and a damping device (ROSE™, Argon Medical Devices, TX, USA) on the frequency characteristics of pressure-transducer kits. The FloTrac sensor kit (Edwards Lifesciences, CA, USA) and the DTXplus transducer kit (Argon Medical Devices) were prepared with planecta ports, and their frequency characteristics were tested with or without ROSE™. The natural frequency and damping coefficient of each kit were obtained using frequency characteristics analysis software and evaluated by plotting them on the Gardner’s chart. By inserting a planecta port, the natural frequency markedly decreased in both the FloTrac sensor kit (from 40 to 22 Hz) and the DTXplus transducer kit (from 35 to 22 Hz). In both kits with one planecta port, the damping coefficient markedly increased by insertion of ROSE™ from 0.2 to 0.5, optimising frequency characteristics. In both kits with two planecta ports, however, the natural frequency decreased from 22 to 12 Hz. The damping coefficient increased from 0.2 to 0.8 by insertion of ROSE™; however, optimisation was not achieved even by ROSE™ insertion. Planecta ports decrease the natural frequency of the kit. ROSE™ is useful to optimise the frequency characteristics in the kits without or with one planecta port. However, optimisation is difficult with two or more planecta ports, even with the ROSE™ device.

## Introduction

A pressure-transducer kit consists of a pressure transducer, three-way stopcocks, and a pressure-resistant tube, and it has specific frequency characteristics such as natural frequency and damping coefficient [1, 2]. The optimal natural frequency and damping coefficient are considered to be more than 16 Hz and from 0.5 to 0.7, respectively [3, 4]. A decrease in the natural frequency results in a tendency of arterial intra-arterial pressure wave overshoot. An increase in the damping coefficient usually causes undershoot. The combination of these frequency characteristics affects the monitoring accuracy. Furthermore, frequency characteristics may be affected by the presence of planecta blood sampling ports. The natural frequency of a pressure-transducer kit can decrease very easily, and its waveform can overshoot or undershoot. However, is not easy to optimise the frequency characteristics of a pressure-transducer kit by adjusting the damping coefficient. The Resonance Over-Shoot Eliminator device (ROSE™, Argon Medical Devices, TX, USA) is prepared as a built-in pre-calibrated resistor to optimise the damping coefficient of the pressure-transducer kit [5, 6]. Some surgical procedures may require the addition of a few planecta ports to a pressure-transducer kit. The aim of the present study was to investigate the effect of planecta ports and the ROSE™ device on the frequency characteristics of blood pressure-transducer kits, as well as the usefulness of the ROSE™ device.

## Methods

### Pressure-transducer kits

**Fig. 1:**
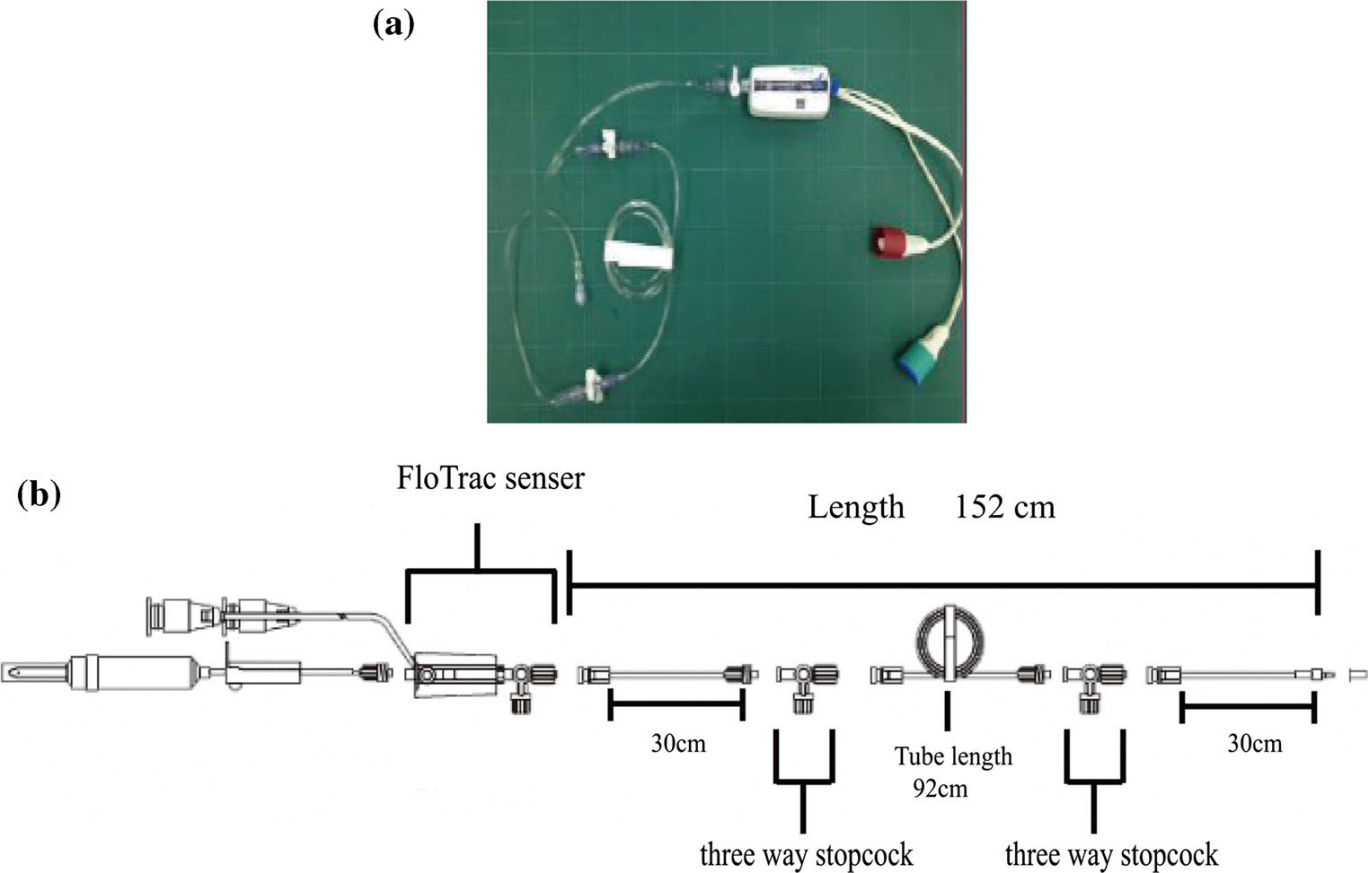
FloTrac sensor kit (Edwards Lifesciences, CA, USA). **a** MHD6S. **b** Component of the MHD6S

**Fig. 2:**
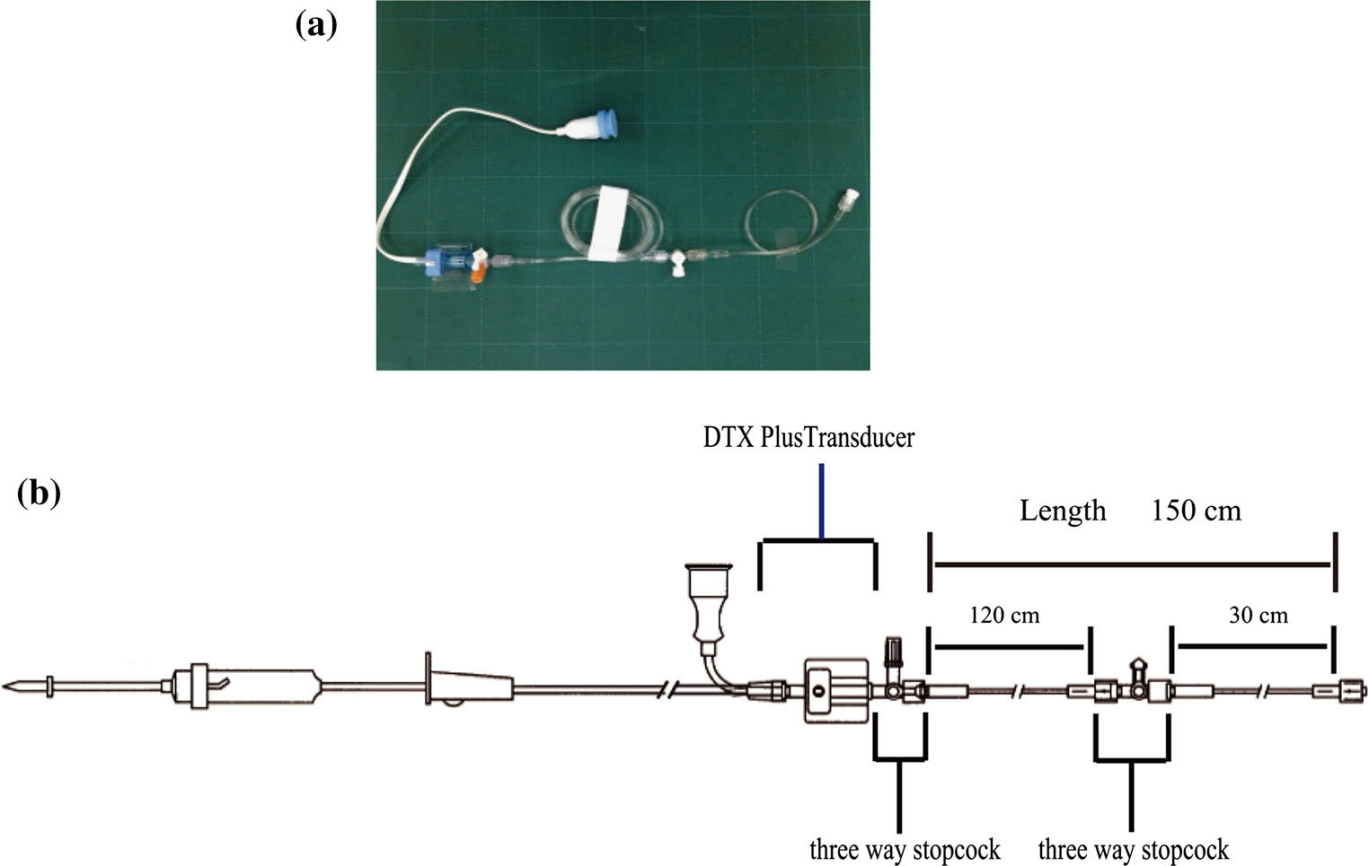
DTXplus transducer kit (Argon Medical Devices, TX, USA). **a** DT4812 J. **b** Component of the DT4812 J

**Fig. 3:**
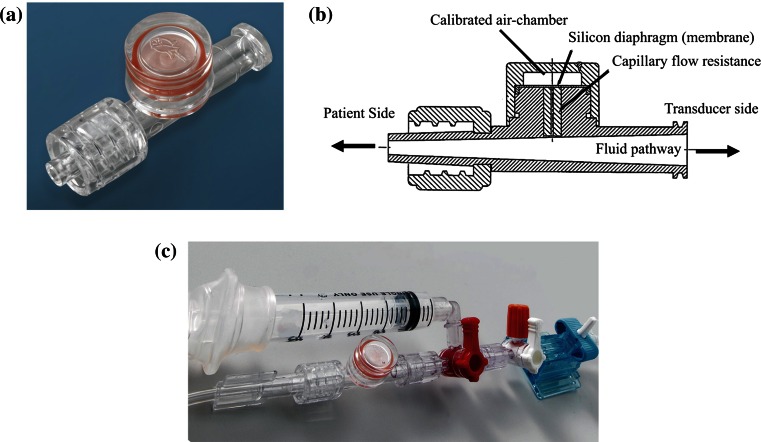
The ROSE™ damping device (Argon Medical Devices, TX, USA) (**a**, body; **b**, internal structure; **c** mounted to kit circuit)

Figure 1 shows the pressure transducer circuits used in the study: the FloTrac sensor kit (MHD6S, Edwards Lifesciences [ED], CA, USA) and the DTXplus transducer kit (DT4812J, Argon Medical Devices [AMD]). Each kit was prepared with planecta ports, and their frequency characteristics were tested with or without ROSE™. The MHD6S, 150 cm in length, is the most basic FloTrac sensor kit, and its circuit contains two L-shaped three-way stopcocks and no planecta port (Fig. 1). In the present study, the three-way stopcocks were replaced with one or two flat planecta ports (AMD). The DT4812J, 150 cm in length, is the most basic pressure transducer kit produced by AMD, and has one L-shaped three-way stopcock and no planecta port (Fig. 2). Additionally, the three-way stopcock of this kit was replaced with one or two flat planecta ports (AMD). The ROSE™ damping device was used as a resistor to cancel circuit vibrations (Fig. 3a–c).

### Analysis of frequency characteristics

**Fig. 4:**
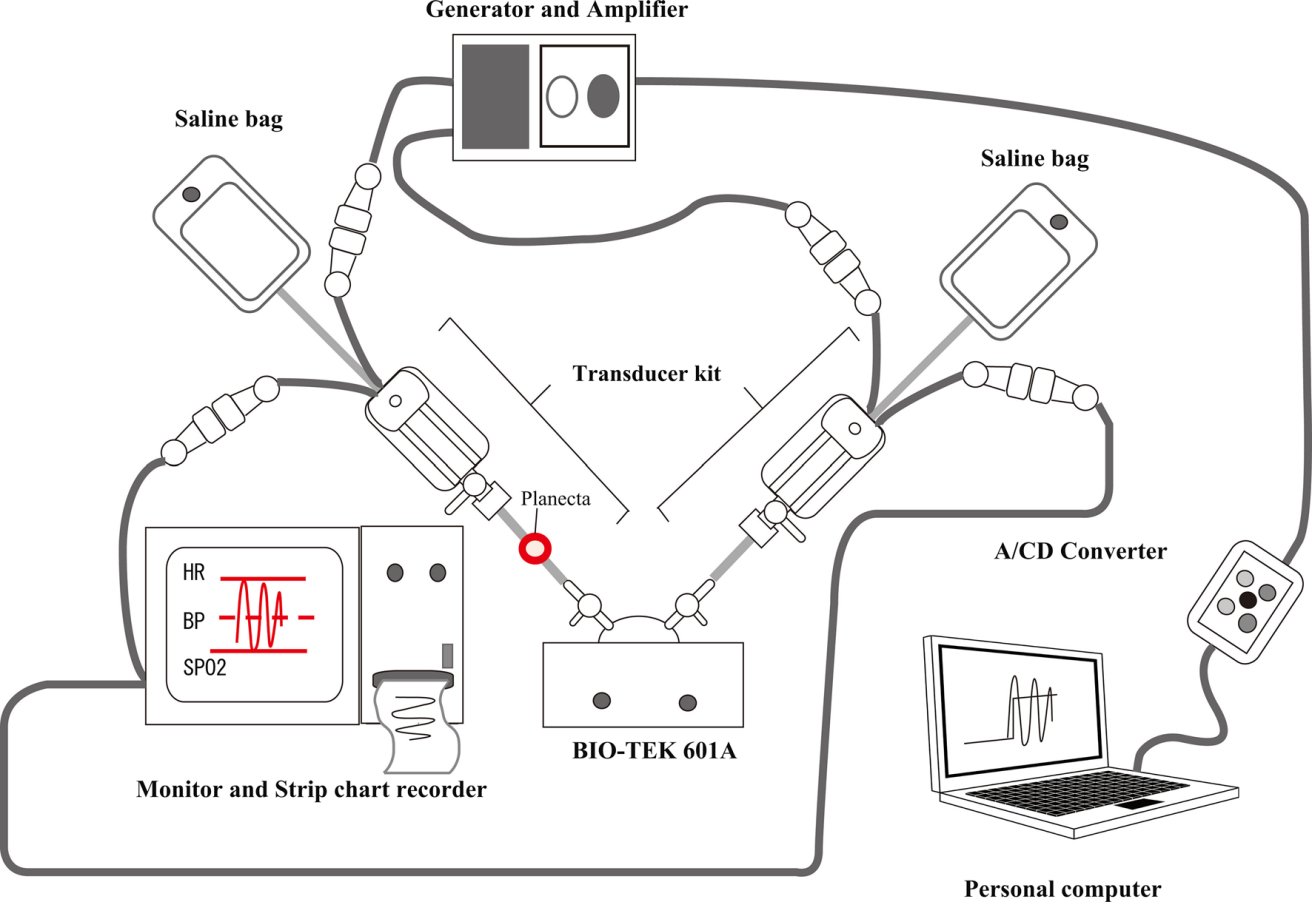
The measurement system of the frequency characteristics and waveforms. The frequency characteristics and waveforms of each circuit were analysed using these measurement devices

**Fig. 5:**
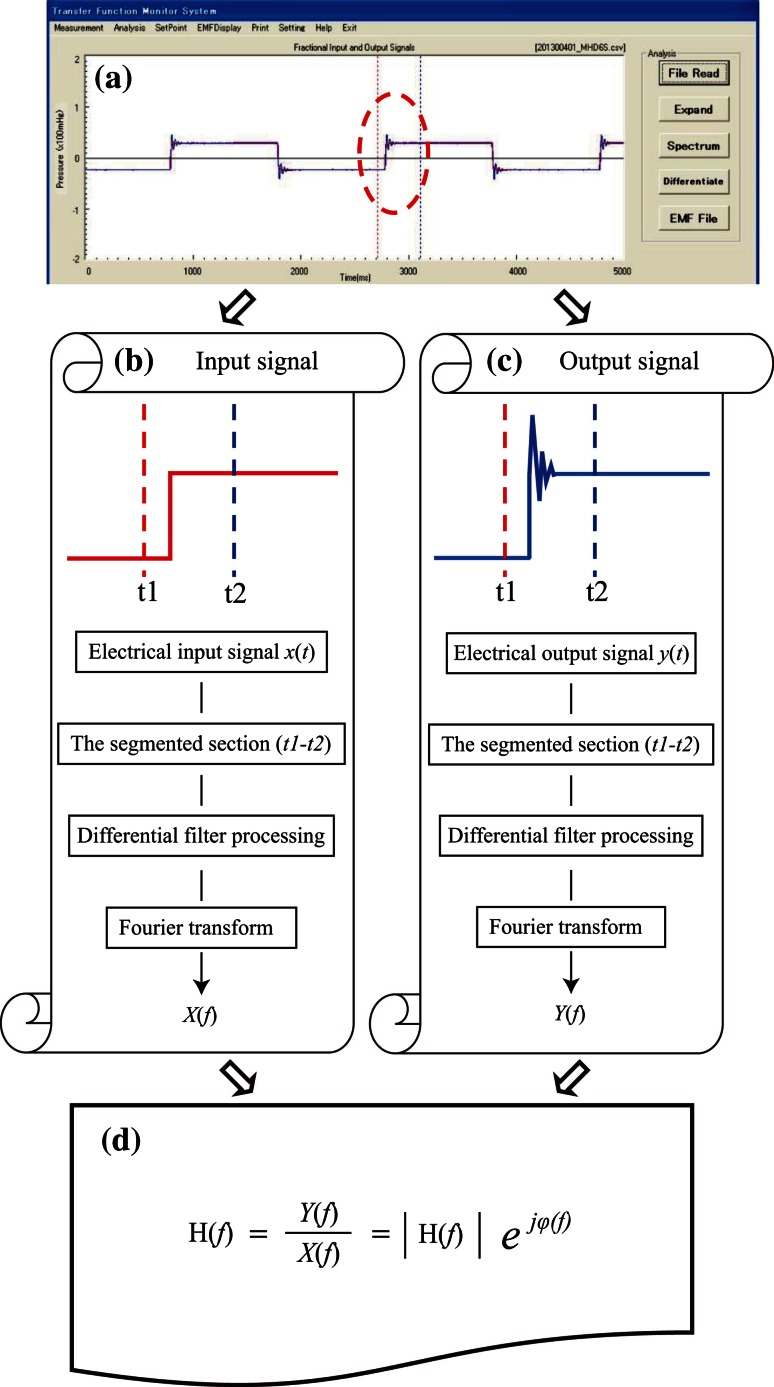
Calculating methods of the frequency characteristics and transfer coefficient. **a** The natural frequency and damping coefficient of each circuit were analysed using a Transfer Function Monitor (Argon Medical Devices, TX, USA) installed in a personal computer. **b** Electrical signal processing of input pressure signal wave. **c** Electrical signal processing of output pressure signal wave. **d** General formulas for the frequency characteristics. These formulas were used to obtain the frequency characteristics of each circuit. |H(*f*)|: the absolute value of H(*f*) and/or amplitude spectrum, *φ(f)*: phase spectrum, *j*: complex number, *e*: napier’s constant

The measuring devices used in the present study are illustrated in Fig. 4. The methods have been previously published by Watanabe et al. [7]. The square wave test was used in this study. A square wave signal was generated with the BIO-TEK 601A (BIO-TEK, Indianapolis, IN, USA). The step response was also used to derive the frequency characteristics of each circuit as shown in Fig. 5. The natural frequency and damping coefficient of each circuit were calculated using a Transfer Function Monitor (AMD, Fig. 5a). Input signals (*x*[*t*]) were segmented in the section from t1 to t2. Differential filter processing and Fourier transform were performed in the segmented section (t1–t2). Waveform *X(f)* was derived as a result of these series of processes (Fig. 5b). Output signals (y [*t*]) were segmented in the section from t1 to t2. Differential filter processing and Fourier transform were performed in the segmented section (t1–t2). Waveform *Y*(*f*) was derived as a result of these series of processes (Fig. 5c). Figure 5d shows the transfer function and spectrum. H(*f*) is a spectrum of H(*t*). Formula (1) shows the general formula of the spectrum. H(*f*) not only indicates transfer function, but also describes frequency characteristic curves.

1 $${\text{H}}\left( f \right) = \frac{Y(f)}{X(f)} = \left| {{\text{H}}\left( f \right)} \right|e^{j\varphi \left( f \right) }$$

Subsequently, the frequency characteristics of each circuit were evaluated by plotting them on Gardner’s chart. Frequency characteristics of each circuit were assessed using Gardner’s chart [8]. Gardner’s chart, which shows the recommended range of natural frequency and damping coefficient, was used to plot the frequency characteristics of each kit.

### Analysis of waveforms

**Fig. 6:**
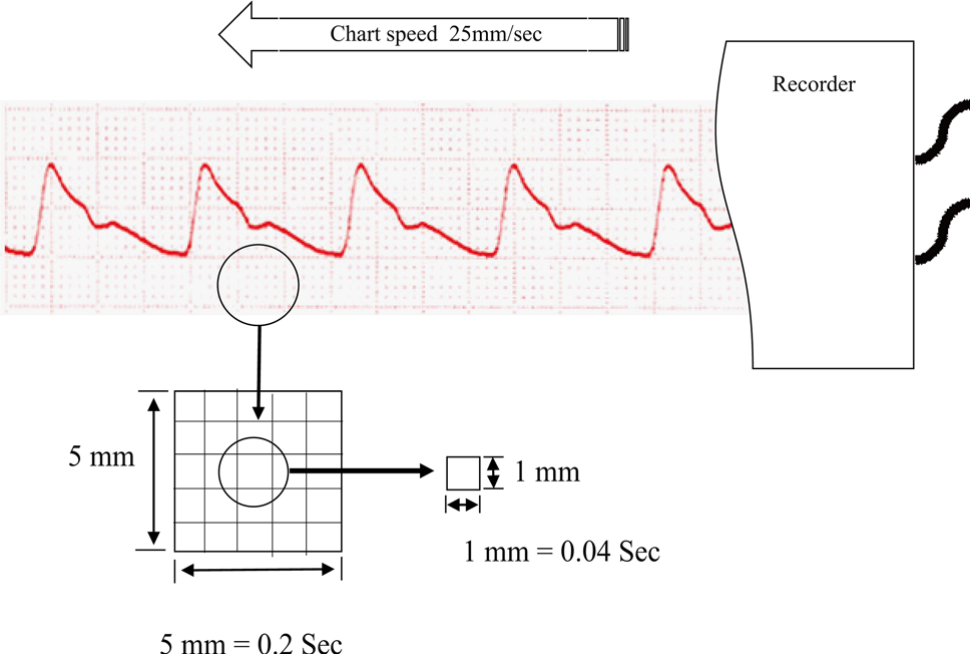
The waveform tracing system. The waveforms of each circuit were monitored by the BSM-4101 (Nihon Kohden, Tokyo, Japan). The monitored waveforms were recorded and traced on 1-mm square charts using a strip chart recorder. The chart speed was 25 mm/s

BIO-TEK 601A was used as an artificial blood pressure source. This device could provide typical artificial radial pressure waves. The pressure waveforms obtained from each pressure-transducer circuits were characterised using artificial radial pressure waveforms. The waveforms of each kit were monitored by the medical monitor (BSM-4101, Nihon Kohden, Tokyo, Japan). The waveforms monitored by the BSM-4101 were plotted on 1-mm square charts (Fig. 6). These plotted waveforms on the charts were scanned and converted to digital images. The digital images were processed, overlaid, and compared by using ImageJ software (http://rsb.info.nih.gov/ij/) and a personal computer [9].

## Results

**Fig. 7:**
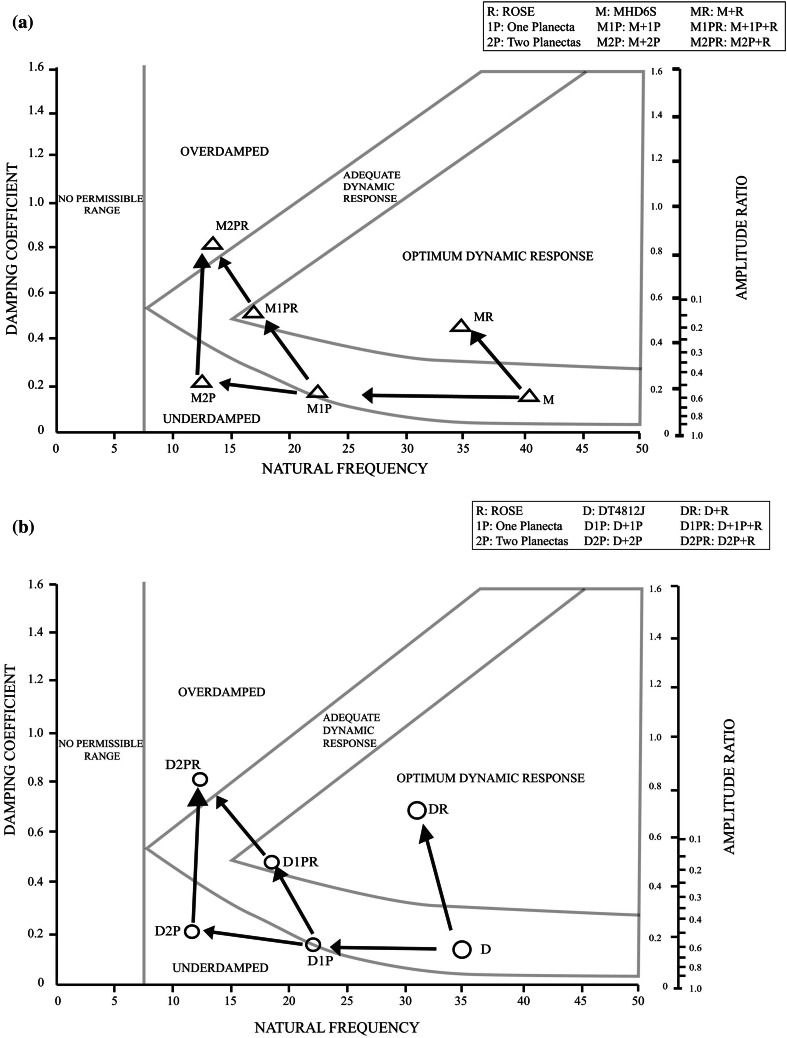

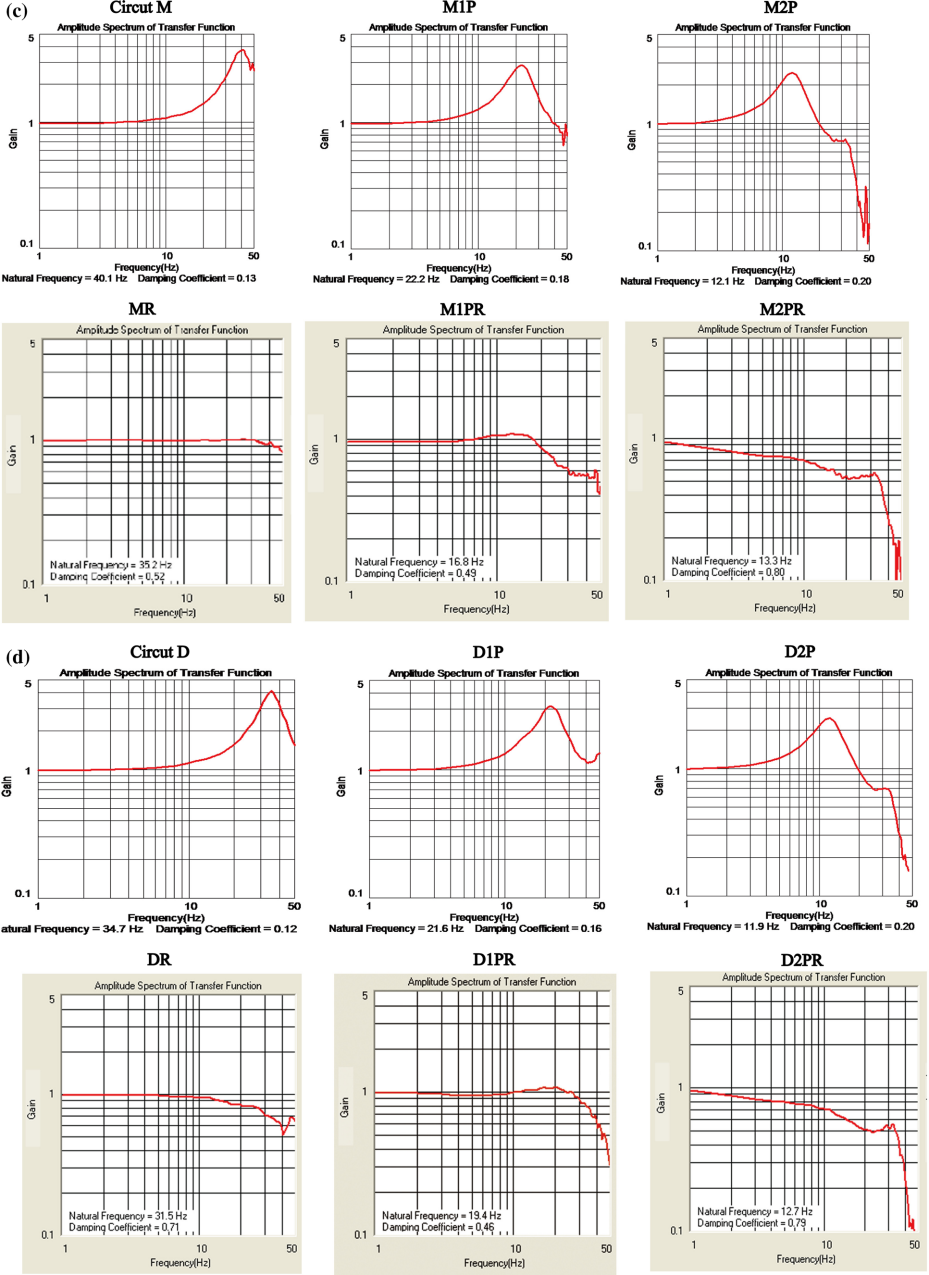
Frequency characteristics of the kits on Gardner’s chart. In the underdamped area (*lower left*), the pressure waveform shows overshoot. In the overdamped area (*upper left*), the pressure waveform shows reduction of the peak (*undershoot*). **a** Frequency characteristics of the Edwards Lifesciences (ED) (CA, USA) circuits. M, 1P, and 2P denote MHD6S, one planecta port, and two planecta ports, respectively. R shows ROSE™ (Argon Medical Devices, TX, USA). M1P is the circuit which incorporated one planecta port in M. M2P is the circuit which incorporated two planecta ports in M. MR consists of M and ROSE™. M1PR consists of M1P and ROSE™. M2PR consists of M2P and ROSE™. **b** Frequency characteristics of the Argon Medical Devices (AMD) circuits. D, 1P, and 2P denote DT4812J, one planecta port, and two planecta ports, respectively. R shows ROSE™. D1P is the circuit which incorporated one planecta port in D. D2P is the circuit which incorporated two planecta ports in D. D1PR consists of D1P and ROSE. D2PR consists of D2P and ROSE™. DR consists of circuit D and ROSE™. **c** Amplitude frequency response curves (amplitude spectrum of transfer function) of ED circuits. The upper graphs show the amplitude frequency response curves of M, M1P, and M2P circuits. The lower graphs show the amplitude frequency response curves of MR, M1PR, and M2PR circuits. **d** Amplitude frequency response curves (amplitude spectrum of transfer function) of AMD circuits. The upper graphs show the amplitude frequency response curves of D, D1P, and D2P circuits. The lower graphs show the amplitude frequency response curves of DR, D1PR, and D2PR circuits

Circuit M denotes the basic FloTrac circuit MHD6S. For circuit M1P with one planecta port, the natural frequency markedly decreased from 40.1 to 22.2 Hz, and for circuit M2P with two planecta ports, the natural frequency further decreased to 12.1 Hz. The inclusion of planecta ports, therefore, substantially reduced the natural frequency, making the circuits slightly underdamped. For circuit MR, which consisted of circuit M and ROSE™, the damping coefficient markedly increased from 0.13 to 0.52 for circuit M; for M2PR with two planecta ports and ROSE™, the damping coefficient increased from 0.2 to 0.8 for circuit M2P. Therefore, an increase in the number of planecta ports tended to lead to overdamping of ED circuits connected to ROSE™. Frequency characteristics of the ED circuits were plotted on Gardner’s chart (Fig. 7a).

For circuit DR, which consisted of circuit D and ROSE™, the damping coefficient increased markedly from 0.12 to 0.71 for circuit D. For circuit D1PR consisting of circuit D1P and ROSE™, the damping coefficient also increased from 0.16 to 0.46 for circuit D1P. In addition, for circuit D2PR consisting of circuit D2P and ROSE™, the damping coefficient markedly increased from 0.2 to 0.79 for circuit D2P. Therefore, an increase in the number of planecta ports tended to lead to overdamping of AMD circuits connected to ROSE™. Frequency characteristics of the AMD circuits were plotted on Gardner’s chart (Fig. 7b). Circuit D is the basic AMD circuit DT4812J. For circuit D1P with one planecta port, the natural frequency markedly decreased from 34.7 to 21.6 Hz; for circuit D2P, the natural frequency further decreased to 11.9 Hz because it contained an additional planecta port. The inclusion of planecta ports, therefore, substantially reduced the natural frequency, making the circuits slightly underdamped.

The amplitude and phase spectrum of transfer functions (frequency response curves) were demonstrated (Fig. 7c, d). Figure 7c, d shows frequency response curves of ED and AMD circuits. The vertical axis shows the input/output ratio (Gain), and the cross axis shows the natural frequency. The ideal input/output ratio is almost 1 and reveals a flat amplitude spectrum curve.

**Fig. 8:**
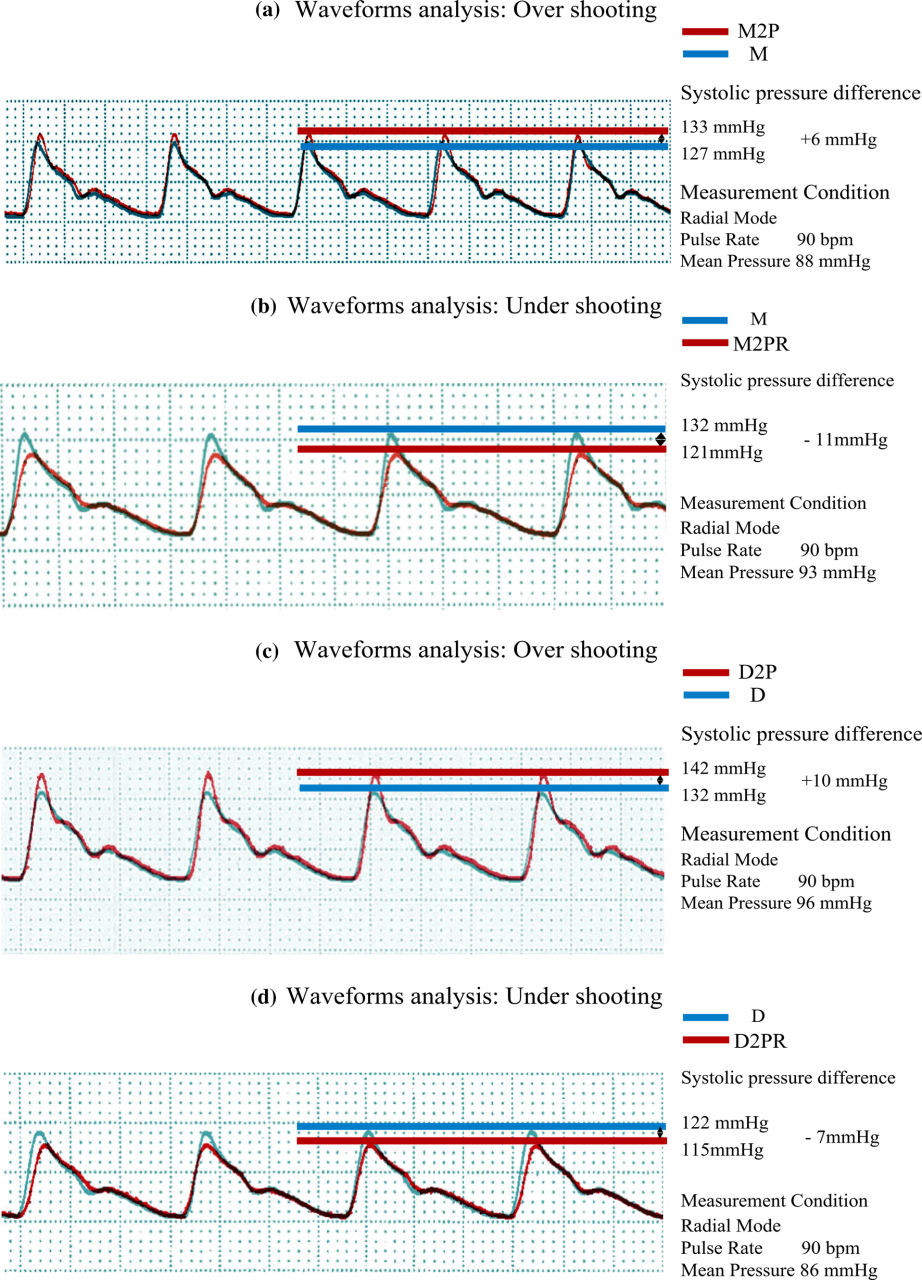
Analyses of the waveforms. **a** The pressure difference in the systolic phase was +6 mmHg. The M2P waveform (*red line*) demonstrated overshoot. **b** The pressure difference in the systolic phase was −11 mmHg. The M2PR waveform (*red line*) demonstrated undershoot. **c** The pressure difference in the systolic phase was +10 mmHg. The D2P waveform (*red line*) demonstrated overshoot. **d** The pressure difference in the systolic phase was −7 mmHg. The D2PR waveform (*red line*) demonstrated undershoot

Figure 8 illustrates a comparison of pressure waveforms. Circuits M2P and M revealed significant overshoot in the M2P waveform (Fig. 8a, red), whereas the same analysis between circuits M2PR and M showed significant undershoot in the M2PR waveform (Fig. 8b, red). Circuits D2P and D revealed significant overshoot in the D2P waveform (Fig. 8c, red), whereas the same analysis in circuits D2PR and D showed significant undershoot in the M2PR waveform (Fig. 8d, red). By inserting a planecta port, the natural frequency markedly decreased in both the FloTrac sensor kit (from 40.1 to 22.2 Hz) and the DTXplus transducer kit (from 34.7 to 21.6 Hz). In both circuits with one planecta port (M1P and D1P), the damping coefficient markedly increased by insertion of ROSE™ (M1P: from 0.18 to 0.49, D1P: from 0.16 to 0.46), without greatly changing the frequency characteristic. However, by inserting two planecta ports, the natural frequency decreased in M2P (from 22.2 to 12.1 Hz) and D2P (from 21.6 to 11.9 Hz). The damping coefficient increased in M2PR (from 0.2 to 0.8) and D2PR (from 0.2 to 0.79) by insertion of the ROSE™ device. Both kits with two planecta ports and ROSE™ showed a tendency of overdamping. In a kit with two planecta ports, actual pressure waveforms changed from overshoot to undershoot by insertion of ROSE™.

## Discussion

A planecta port is useful to avoid babbling and/or bacterial contamination [10], but it decreases the natural frequency of the kit. ROSE™ is useful to optimise the frequency characteristics of the kits with no or one planecta port [11, 12]. However, the kit with two planecta ports showed underdamping, and when ROSE™ was inserted, it showed overdamping due to the very low natural frequency. Two or more planecta ports, therefore, should not be used for accurate monitoring.

The combined use of a pressure transducer kit and the planecta closed injection system is recommended for preventing infection, enabling technical simplicity, and excluding air. However, a large number of planecta ports resulted in a large reduction of the natural frequency, resulting in underdamping of kit circuits and waveform undershoot. The inclusion of ROSE™ considerably increased the damping coefficient of the circuits and subsequently reduced underdamping. Yet, overdamping was still noted when two planecta ports and ROSE™ were combined.

Therefore, it may be necessary to avoid the use of more than two planecta ports in a kit circuit. In fact, AMD Co. in Europe does not recommend the use of more than two planecta ports. In general, the acceptable range of damping coefficients is 0.5–0.7, and the ideal natural frequency value is >20 Hz, although >16 Hz is also acceptable [3, 4, 13].

As shown in the present study, ROSE™ provides a higher damping coefficient without a reduction in frequency range. ROSE™ does not require damping adjustment because of the device’s built-in pre-calibrated resistor. Furthermore, because ROSE™ is ready-to-use and can be directly mounted to a pressure transducer kit, it is extremely useful in clinical practice. This study was performed using artificial blood pressures. Further study should confirm our results in clinical cases.

At heart rates of 60–120 beats-per-minute, the fundamental frequency is 1–2 Hz in arterial pressure signal, and this fundamental frequency must be transmitted up to 8 times (8–16 Hz) without distortion [13]. The resonant frequency of putting a fluid-filled cannula/tubing system in between the artery and the transducer was around 16 Hz (transducers without tubing had resonant frequencies of well over 100 Hz) [13]. Frequency response curves of M2P and D2P are demonstrated in Fig. 7c, d. The peak of both frequency response curves was around 12 Hz. The result of these measurements indicated that M2P and D2P circuits were producing a resonance phenomenon. In contrast, the Gain of M2PR and D2PR circuits had been clearly decreasing in between 1 and 16 Hz. These results meant that the frequencies of M2PR and D2PR circuits were strongly suppressed by ROSE™. As it was previously mentioned, the ideal Gain is almost 1. The circuits with an ideal Gain were MP, M1PR, DR, and D1R in between 1 and 16 Hz (Fig. 7c, d). The circuits with acceptable Gain were M, M1P, D, and D1P in between 1 and 16 Hz.

In conclusion, each pressure transducer kit has an appropriate combination of natural frequency and damping coefficient. Taking into account the frequency characteristics of the circuits, pressure transducer kits should be used with a ROSE™ device to ensure an optimal combination of natural frequency and damping coefficient.
